# Effect of pulmonary rehabilitation on lung cancer surgery outcomes: a matched-case analysis

**DOI:** 10.1186/s13741-025-00510-2

**Published:** 2025-03-25

**Authors:** Matar Alzahrani, Rajnikant Mehta, Salma Kadiri, Saffana Algaeed, Aya Osman, Mohammed Alsanad, Joan Duda, Fang Gao, Babu Naidu

**Affiliations:** 1 College of Applied Medical Sciences, King Saud Bin Abdulaziz University for Health Sciences, Jeddah, Saudi Arabia; 2 Institution of Inflammation and Aging, University of Birmingham, Birmingham, UK; 3 King Abdullah International Medical Research Center, Jeddah, Saudi Arabia; 4 Wolfson Institute of Population Health, University of London, Mile End, Queen Mary UK; 5 Thoracic Surgery, University Hospitals Birmingham NHS Foundation Trust, Birmingham, UK; 6 King Saud University, Riyadh, Saudi Arabia; 7 Warwick Clinical Trials Unit, University of Warwick, Coventry, UK; 8 School of Sport, Exercise and Rehabilitation Sciences, University of Birmingham, Birmingham, UK

**Keywords:** Pulmonary rehabilitation, Prehabilitation, Postoperative pulmonary complications, Quality of life, Propensity score analysis, Hospital length of stay, Rehabilitation programs

## Abstract

Pulmonary rehabilitation programs for COPD patients are extensively accessible throughout the UK and have demonstrated efficacy in enhancing outcomes, including recovery from exacerbations. Numerous lung cancer surgery patients possess COPD, and the surgery may be regarded as a definitive aggravation of COPD. It is ambiguous in practical application whether referral to pulmonary rehabilitation programs enhances surgical and patient-reported results. We want to address this topic by conducting a propensity-score analysis (PSA) of participants in an enriched cohort trial.

**Methods** An enriched cohort research was conducted, providing rehabilitation both pre- and post-surgery pragmatically through local pulmonary rehabilitation providers for patients undergoing lung cancer resection, and compared to a contemporaneous control group receiving standard treatment. The study enrolled 873 participants (pulmonary rehabilitation (PR) *n* = 135, non-intervention or control (NG) *n* = 738). Regression analyses for exposed and unexposed matching, effect estimation, and standard error estimations were conducted.

**Results** A total of 114 participants were matched PR (*n* = 57) and NG (*n* = 57). The multivariate-linear regression indicated a reduction in length of stay (LOS) of 0.2 days of LOS compared to the usual care group (EE = − 0.20), and that reduction could potentially go up to 1.8 days (95% CI = − 1.8–1.6). The multivariate log-binomial regression revealed that PR had a reduction of 60% postoperative pulmonary complications (PPC) rate (EE = − 0.60, 95% CI = − 1.8–0.5). Lastly, the multivariate-linear regression showed an improvement in quality of life 6 weeks and 6 months after surgery (QoL) in patients in PR, especially in the physical functioning score in which an improvement of 6.6% was noted for the PR group compared to the NG group following surgery (EE = 6.6). Conclusion Participation in “real world” pulmonary rehabilitation prior to and following surgery seems to yield improved patient and clinical results post-lung cancer surgery. Nonetheless, prompt access to pulmonary rehabilitation may be a significant challenge following COVID.

**Supplementary Information:**

The online version contains supplementary material available at 10.1186/s13741-025-00510-2.

## Introduction

Major complications occur in up to 20% of patients undergoing chest or abdominal surgery, with up to 13% of patients developing postoperative pulmonary complications (PPC) following curative lung cancer surgery, which is often exacerbated by underlying COPD (Smith et al. 2012; Taylor et al. 2023; Jeganathan et al. 2022). PPC significantly increases mortality, intensive therapy unit (ITU) admissions, length of hospital stay, and 30-day hospital readmissions, and its occurrence across various surgeries highlights the potential for cost savings through improved rehabilitation (Myles et al. 2020; STARSurg Collaborative. Impact of postoperative non-steroidal anti-inflammatory drugs on adverse events after gastrointestinal surgery. 2014; Jones et al. 2019; Katsura et al. 2013; Bradley et al. 2013).

As surgery is increasingly performed on older and less-fit patients, structured post-surgical programs, including physical activity, nutritional guidance, and psychological interventions, have shown effectiveness in reducing mortality, improving quality of life (QoL), and preventing hospital readmissions (Bowel cancer statistics 2020; McCann et al. 2019; Arora et al. 2018). These successes have sparked interest in prehabilitation as a proactive strategy to enhance surgical outcomes and further reduce complications (McCann et al. 2019; Arora et al. 2018).

Prehabilitation before surgery has been recognized as a critical step to improve postoperative outcomes. It enhances an individual’s functional capacity to withstand the stress of major surgery (Banugo and Amoako 2017). Prehabilitation can lead to significant improvements in patients’ physical fitness, mental well-being, and overall readiness for surgery (Tew et al. 2020). The American College of Surgeons highlights that prehabilitation helps enhance the functional capacity of patients before surgery, making them more resilient to postoperative inactivity and decline (The American College of Surgeons (n.d.)). This multimodal approach includes exercise training, nutritional support, and psychological interventions, which collectively help in reducing perioperative complications and improving recovery times (Banugo and Amoako 2017).

Access to prehabilitation services in the UK and globally remains limited despite its recognized benefits in improving surgical outcomes. In the UK, a significant proportion of patients are not offered prehabilitation due to inconsistent implementation across health services (Wade-Mcbane et al. 2023). For example, a study revealed that while the need for prehabilitation is widely acknowledged, only a small percentage of patients actually receive these services (Wade-Mcbane et al. 2023). This gap is often attributed to a lack of resources, standardized protocols, and sufficient training for healthcare providers (Myles et al. 2020). Globally, the situation is similar. For instance, a survey of thoracic surgeons in Australia found that although there is a high perceived need for prehabilitation, only a small fraction of patients had access to these services (Grocott et al. 2023). This disparity highlights the broader issue of unequal access to prehabilitation across different countries and healthcare systems (Grocott et al. 2023).

In response to this rising need to enhance outcomes, prehabilitation may have significant relevance for frail and elderly populations awaiting an elective major operation (Bowel cancer statistics 2020). There is currently no established pathway for engaging these patients in improving their health as they wait for their major surgical interventions (Arora et al. 2018). Furthermore, in real-world practice, it is uncertain whether referral to pulmonary prehabilitation classes improves surgical and patient-reported outcomes. Using real-world data improves generalizability by including diverse patient populations often excluded from randomized controlled trials (RCTs) and better reflects routine clinical practice, capturing real-world treatment effectiveness, adherence, and patient behaviors (Sherman et al. 2016; Califf 2016). Recent studies have demonstrated that pulmonary rehabilitation significantly reduces postoperative complications, particularly pulmonary complications, and improves recovery in lung cancer surgery patients, supporting its potential role in this context (Wang et al. 2023; Mao et al. 2021). Therefore, whether referral to pulmonary rehabilitation classes could be an alternative for patients who are waiting for lung cancer surgery to reduce the incidence or severity of postoperative complications should be investigated (McCann et al. 2019; Arora et al. 2018). By performing a propensity-score analysis (PSA), we aim to investigate whether the prehabilitation program, which is a referral to pulmonary rehabilitation classes, had an impact on perioperative care compared to the usual care, no prehabilitation, on patients who underwent lung cancer surgery.

## Methods

### Study design

This study is a prospective, longitudinal, single-center study. The Institutional Review Board approved the study (REC number: 10/H1208/41).

### Participants and procedures

This enriched cohort study involved consecutive recruitment of patients undergoing lung cancer resection at University Hospital Birmingham NHS Foundation Trust by offering rehabilitation pre- and post-surgery pragmatically by local providers compared to a contemporaneous control group who just had usual care. Informed consent was obtained from all participants, and quality of life (QoL) measures were self-reported by all participants following lung surgery. Data was collected prospectively from patients’ medical records including length of stay (LOS) and postoperative pulmonary complications (PPC) using the Melbourne group scale (MGS) (Lugg et al. 2016).

The study recruited 873 participants who underwent lung cancer surgery from 2010 to 2020. Participants were allocated to two groups: pulmonary rehabilitation (PR) (*n* = 135) and non-intervention or control (NG) (*n* = 738). Participants in the PR offered rehabilitation pre- and post-surgery pragmatically by local providers, and participants in the NG received usual care. The inclusion criteria were above the age of 18 years, undergoing lung cancer curative resection, able to provide written informed consent, and before surgery at the time of consent. The exclusion criteria were unable to provide written informed consent. Participants were recruited at least 2 weeks prior to surgery and were followed up to 5 months after surgery in both groups.

### PR program

The participants were recruited from 11 hospitals across the UK, of which only three—Walsall Manor, Heartlands, and Worcester—had pulmonary rehabilitation programs in place (British Thoracic Society Standards of Care Committee. BTS guideline on pulmonary rehabilitation in adults 2013). Patients in the PR group were enrolled in a 12-week pulmonary rehabilitation program at these three hospitals, commencing before their lung cancer surgery. Each patient attended at least two sessions prior to their surgery. Worcester offered a community-based program, while Heartlands and Walsall Manor hospitals provided in-hospital, class-based programs. These programs aligned with the British Thoracic Society’s guidelines, which recommend a minimum 6-week PR program with twice-weekly supervised sessions, incorporating tailored aerobic and resistance exercise training, structured education, and a multidisciplinary team approach to improve functional capacity, symptom management, and quality of life for patients with chronic respiratory conditions (British Thoracic Society Standards of Care Committee. BTS guideline on pulmonary rehabilitation in adults 2013).

### Outcome measures

The primary outcome measures were hospital length of stay (LOS), postoperative pulmonary complications (PPC) defined using the MGS (Lugg et al. 2016), and quality of life (QoL). The LOS and PPC were extracted from the medical records. The QoL for the participants was measured in baseline before surgery, 6 weeks after surgery, and 6 months after surgery. The EORTC QLQ-C30 is a standardized questionnaire designed to measure the quality of life in cancer patients. It evaluates three main domains: functional (physical, role, emotional, cognitive, and social aspects); symptom (issues such as fatigue, pain, nausea, and financial difficulties); and global health, which provides an overall perspective on health and quality of life (Aaronson et al. 1993). Scores are derived using a 0–100 scale for clarity, with raw scores averaged for each domain and linearly transformed for final interpretation. The tool consists of 30 items organized into multi-item and single-item scales. The QoL domains’ scores were interpreted based on the clinically meaningful difference defined as a change of 10 units.

For functional scales and the global health status/QoL scale, higher scores indicate better functioning and quality of life (Aaronson et al. 1993). Conversely, for symptom scales, higher scores indicate greater symptom severity or more problems (Aaronson et al. 1993). These standardized scores facilitate comparison across different studies and patient populations, allowing clinicians and researchers to assess and monitor patients’ quality of life effectively (Fayers et al. 2001).

### Matching, covariates, and statistical analysis

To create a comprehensive and current database, it was essential to clean the existing data and collect new data prospectively from patients’ electronic medical records. To address this challenge, research fellows have been gathering data and building multiple databases over the last decade. Matching was then performed using propensity score analysis (PSA). PSA is a statistical technique used to estimate the effect of a treatment, policy, or intervention by accounting for covariates that predict receiving the treatment (Austin 2011). This method is particularly useful in observational studies where random assignment is not feasible (Austin 2011). By using PSA, researchers aim to reduce selection bias and simulate a randomized controlled trial. The key concept is the propensity score, which is the probability of a unit (e.g., a person) receiving the treatment given their observed characteristics. PSA is widely used in fields such as epidemiology, economics, and social sciences to draw causal inferences from non-experimental data.

**Fig. 1:**
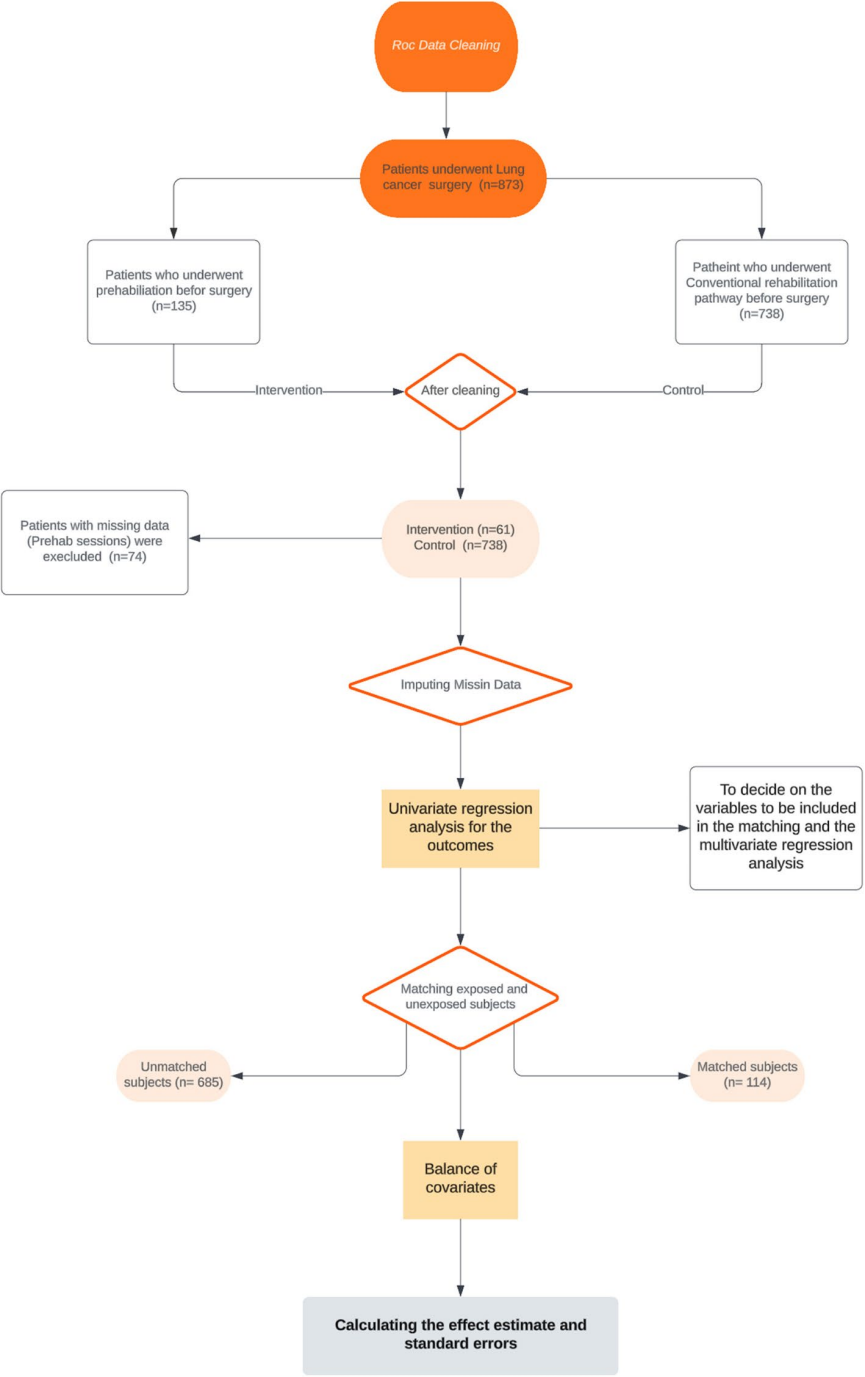
PSA steps flowchart for evaluating pulmonary rehabilitation impact on lung cancer surgery patients

Due to the missing values within the data set, as presented in Fig. 1, imputation techniques were applied using RStudio version 2024.04.2 + 764 before moving to univariate regression for the outcomes. The data imputation approach that was used is the MICE package installed in R (Buuren and Groothuis-Oudshoorn 2011). The package creates multiple imputations (replacement values) for multivariate missing data. The approach relies on fully conditional specification, wherein each incomplete variable is imputed using an individual model. The MICE algorithm can impute combinations of continuous, binary, unordered categorical, and ordered categorical data. Furthermore, MICE is capable of imputing continuous two-level data while ensuring consistency among imputations by passive imputation. Numerous diagnostic plots are utilized to evaluate the quality of the imputations.

**Fig. 2:**
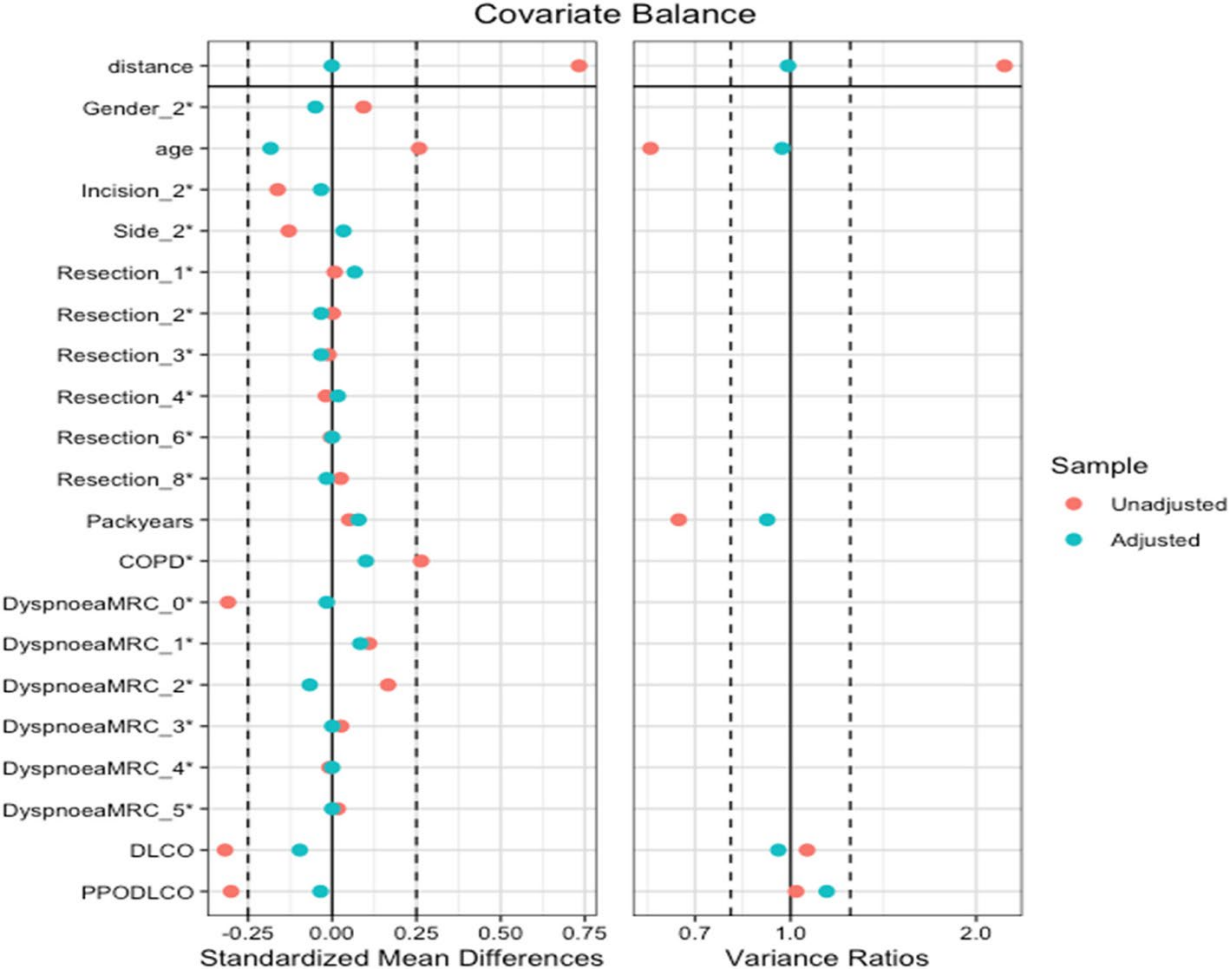
Covariates balance assessment before and after propensity score matching

Figure 1 summarises the propensity score analysis (PSA) steps. After carrying out the regression analysis for the outcomes, the following covariates were included in the matching: COPD, gender, age, incision, side, resection, MRC dyspnoea, DLCO, and packyears. After deciding on the variables to include and performing the regression for each outcome, the next steps in PSA are to match exposed and unexposed subjects, then check the balance of covariates in the exposed and unexposed groups after matching, and finally calculate the effect estimate and standard errors with this match population. After matching, it is essential to check the balance of covariates between the treated and control groups. This step ensures that the matching process successfully creates comparable groups (Fig. 2). Figure 2 shows the standardised mean differences for covariates before (red) and after (blue) adjustment in PSA. The two vertical dashed lines represent the thresholds for acceptable balance, typically set at ± 0.1, indicating that covariates with differences within these lines are considered well balanced (Fig. 2).

### Propensity score model

After selecting covariates and performing regression for each subject, the propensity score analysis (PSA) proceeded with matching exposed and unexposed subjects, checking covariate balance post-matching, and calculating effect estimates and standard errors. The matching process was conducted in R using the MATCHiT version 4.6.0 package, with nearest neighbor matching within a calliper of 0.02 to ensure that the propensity scores (PS) of matched pairs were close enough for comparability (Ho et al. 2011). A calliper of 0.02 was chosen to balance precision and confidence in match quality, as values too small or large could compromise matching effectiveness. A 1:1 matching ratio without replacement was used, meaning each unexposed subject was matched to only one exposed subject, enhancing precision by maximizing the use of available subjects.

## Results

### General patient characteristics

A total of 873 patients were recruited from 2010 to 2020 who underwent lung cancer surgery and were included in the statistical analysis (Fig. 1). After data cleaning, 74 patients were excluded from the intervention arm since there was no information about how many prehabilitation sessions they had (Fig. 1). The participants’ baseline characteristics and clinical data are shown in Table 1.

**Table 1:** Baseline characteristics of 799 patients included in the study

Characteristic	Pulmonary rehabilitation (PR) (*n* = 61)	Non-intervention or control (NG) (*n* = 738)
**Patient demographics**		
Age	71 (66, 75)	70 (63, 75)
Gender (male)	26 (43%)	383 (52%)
BMI	25.2 (22.8, 28.5)	26.9 (24.0, 30.3)
**BMI classification**		
Healthy weight	26 (43%)	212 (29%)
Obese	9 (15%)	180 (24%)
Overweight	21 (34%)	296 (40%)
Severely obese	1 (1.6%)	16 (2.2%)
Underweight	4 (6.6%)	34 (4.6%)
**Smoking status**		
Current	9 (15%)	76 (10%)
Ex-smoker	42 (69%)	512 (70%)
Never smoker	10 (16%)	142 (19%)
Pack years	40 (15, 50)	28 (5, 45)
Unknown pack years	9	46
**Lung function**		
FEV_1_ (L)	2.14 (1.70, 2.55)	2.02 (1.56, 2.30)
% FEV_1_	87 (74, 100)	81 (66, 99)
%DLCO	77 (65, 91)	71 (58, 84)
PpoFEV_1_	69 (56, 81)	62 (46, 81)
PpoDLCO	61 (51, 73)	57 (48, 66)
**Surgical incision**		
Open	37 (61%)	327 (44%)
VATS	24 (39%)	409 (56%)
**Surgery side**		
Right	43 (70%)	425 (58%)
**Lobe**		
Upper	37 (63%)	402 (56%)
Middle	2 (3.4%)	44 (6.1%)
Lower	15 (25%)	227 (31%)
Upper bilobe	4 (6.8%)	10 (1.4%)
Lower bilobe	0	5 (0.7%)
Entire lung	1 (1.7%)	26 (3.6%)
**Lung resection**		
Wedge	10 (16%)	129 (17%)
Segmentectomy	2 (3.3%)	23 (3.1%)
Lobectomy	45 (74%)	539 (73%)
Bilobectomy	3 (4.9%)	18 (2.4%)
Sleeve	0	3 (0.4%)
Pneumonectomy	1 (1.6%)	26 (3.5%)
**Comorbidity**		
COPD	27 (44%)	132 (18%)
Hypertension	20 (34%)	316 (46%)
**Other measures**		
MRC dyspnoea score > 2	4 (6.6%)	21 (3%)
**Rehabilitation sessions**		
Pre-surgery	2 (1, 5)	
Post-surgery	0 (0, 2)	
**Perioperative outcomes**		
Postoperative pulmonary complications	7 (11%)	46 (6.2%)
Hospital length of stay (days)	5.0 (4.0, 7.0)	4.00 (3.00, 6.00)

Data are presented as mean (median), standard deviation (interquartile range), or numbers and percentages for categorical data

*FEV*_*1*_ forced expiratory volume for 1 s, *DLCO* diffusing capacity for carbon monoxide, *PpoFEV*_*1*_ or *PpoDLCO* predicted postoperative FEV_1_ or DLCO, *VATS* video-assisted thoracic surgery, *COPD* chronic obstructive pulmonary disease

Table 1 represents baseline demographic and smoking data for 799 patients. The median age is 70 years old, and it can be noticed that around half of the patients are male. It can be seen that the mean BMI for 799 is 26.9.The majority of the patients reported that they had quit smoking before their surgery, while almost a third of the patients in both groups were never smokers. It is apparent that 45% of the patients suffered from hypertension, and 20% had chronic obstructive pulmonary disease (COPD). Regarding postoperative outcomes, only 7% of patients had postoperative pulmonary complications (PPC), and the median length of hospital stay (LOS) was 4 days (Table 1).

### Quality of life scores

Table 2 shows the difference in functional or global health domains at different time points (Supplementary Material: Fig. 3). Clinically, global health has deteriorated from baseline to 6 weeks, generally indicating lower quality of life. Similarly, physical functioning has clinically declined between preoperative 6-week and 5-month scores. Also, emotional functioning has clinically declined between preoperative and 6-week scores; however, there has been a marginal improvement, but not to the point of clinical difference. Regarding the dyspnoea symptom domain clinically, the patients’ scores declined significantly post-surgery at 6 weeks and 6 months compared to that before surgery.

**Table 2:** Comparison between EORTC QLQ-C30 functioning and global health domains, and dyspnoea symptom domain

QoL domain	baseline	6 weeks	6 months
Global health	75 (67, 92)	67 (50, 83)	67 (50, 83)
Physical functioning	93 (80, 100)	**80 (60, 87)**	**80 (60, 93)**
Role functioning	85 (60, 87)	**63 (50, 83)**	**70 (67, 92)**
Emotional functioning	83 (67, 100)	76.6 (67, 92)	83 (67, 100)
Cognitive functioning	85 (60, 87)	80 (60, 87)	83 (67, 100)
Social functioning	85 (60, 87)	83 (67, 100)	85 (60, 87)
Dyspnoea	20.5 ± (25.4)	**41.9 ± (29.6)**	**39.2 ± (29.4)**

Data are presented as mean (median) and standard deviation (interquartile range). Highlighted numbers indicate clinically meaningful differences of 10 units or more

Following propensity score matching, Table 3 shows the difference in functional and global health domains at different time points after matching. Clinically, global health and physical functioning declined from baseline to 6 weeks, indicating a reduction in overall quality of life postoperatively, with a slightly greater decline in the NG group. Role functioning also showed a decrease at 6 weeks, with some recovery at 6 months, though scores remained lower than baseline. Emotional and cognitive functioning showed minor fluctuations over time, with no clinically significant differences between groups. Dyspnoea scores worsened significantly postoperatively in both groups at 6 weeks and remained elevated at 6 months, with NG patients experiencing a greater impact compared to PR patients.

**Table 3:** Comparison between EORTC QLQ-C30 functioning and global health domains, and dyspnoea symptom domain after matching

QoL domain	baseline		6 weeks		6 months	
	PR	NG	PR	NG	PR	NG
Global health	75.42 (20.84)	66.81 (20.61)	62.92 (15.83)	58.06 (20.87)	66.81 (21.89)	61.81 (23.13)
Physical functioning	85.89 (16.56)	78.78 (21.06)	72.22 (17.79)	66.44 (23.19)	74.33 (20.68)	66.56 (25.26)
Role functioning	87.50 (21.18)	79.72 (27.97)	65.28 (28.67)	58.61 (28.87)	68.06 (30.73)	63.33 (34.00)
Emotional functioning	80.00 (18.04)	72.36 (22.21)	75.83 (22.27)	70.51 (24.79)	77.78 (21.19)	75.42 (25.28)
Cognitive functioning	88.33 (17.71)	84.72 (16.89)	80.56 (24.39)	78.61 (20.83)	84.17 (21.13)	78.89 (19.13)
Social functioning	90.28 (18.49)	85.28 (23.59)	73.06 (27.46)	70.56 (29.17)	74.72 (29.67)	76.39 (28.17)
Dyspnoea	21.1 (27.2)	26.6(27.7)	47.20 (28.07)	51.07 (30.71)	42.7 (29.2)	51.6 (31.2)

Data are presented as mean and standard deviation

### Other analyses

Table 4 shows the baseline characteristics of 114 patients included in the study after matching. Once the balance of the matched population is ensured, as indicated in Fig. 2, the final step of the PSA is to calculate the effect estimate (EE) and standard errors (SE) for the outcomes, which are the length of stay (LOS), postoperative pulmonary compilations (PPC), 6 weeks postoperative physical functioning (PF 6W) domain, 6 weeks postoperative dyspnoea (DY 6W), 6 weeks postoperative global health (QoL 6W), and 6 months postoperative global health (QoL 6 M) as indicated in Table 5. Supplementary Material: Fig. 4 points out the forest plot of key outcomes.

**Table 4:** Baseline characteristics of 114 patients included in the study after matching

Characteristic	Pulmonary rehabilitation (PR) (*n* = 57)	Non-intervention or control (NG) (*n* = 57)
**Patient demographics**		
Age	70.48 (7.25)	71.80 (7.36)
Gender (male)	32 (56.7%)	35 (61.7%)
**Smoking status**		
Pack years	32.67 (23.58)	30.82 (24.63)
**Lung function**		
%DLCO	71.38 (18.70)	73.19 (19.13)
PpoDLCO	57.22 (15.41)	57.76 (14.40)
**Surgical incision**		
Open	35 (60%)	33 (56.7%)
VATS	22 (40%)	24 (43.3%)
**Surgery side**		
Right	16 (30%)	14 (26.7%)
**Lung resection**		
Wedge	10 (17.5%)	12 (21.1%)
Segmentectomy	2 (3.5%)	4 (7%)
Lobectomy	41 (71.9%)	37 (64.9%)
Bilobectomy	3 (5.3%)	4 (7%)
Sleeve	0	0
Pneumonectomy	1 (1.8%)	0
**Comorbidity**		
COPD	25 (45%)	19 (34.2%)
**Other measures**		
MRC dyspnoea score > 2	3 (5.3%)	3 (5.3%)

Data are presented as mean (median), standard deviation (interquartile range), or numbers and percentages for categorical data

*DLCO* diffusing capacity for carbon monoxide, *PpoDLCO* predicted postoperative DLCO, *VATS* video-assisted thoracic surgery, *COPD* chronic obstructive pulmonary disease

**Table 5:** Multivariate regression results for the outcomes in the PR group after matching

**Outcomes**	PPC	LOS	PF 6W	DY 6W	QoL 6W	QoL 6 M
**Values**						
EE	− 0.60	− 0.20	6.6	− 0.44	5.1	4.7
SE	0.59	0.82	3.71	0.38	3.34	3.35
Antilog	1.8	-	-	0.64	-	-
95% CI	− 1.8–0.5	− 1.8–1.6	− 0.80–14	− 1.1–0.24	− 1.5–12	− 3.5–13

*CI* confidence interval, *EE* effect estimate, *SE* standard error

In Table 5, it can be noted that the PR group had a reduction of 0.2 days in LOS compared to the usual care group (EE = − 0.20), with the potential reduction extending up to 1.8 days, though the confidence interval also includes the possibility of an increase of 1.6 days (95% CI = − 1.8 to 1.6). In regard to the PPC, the PR group had a reduction of 60% (EE = − 0.60, 95% CI = − 1.8–0.5). There was an indication of a 6.6% improvement in the physical functioning score for the PR group compared to the usual care group following surgery (EE = 6.6). Although it is only 6.6%, the 95% CI indicated that the maximum could be 14 units, which is considered a clinically significant improvement. Also, there was a slight reduction of − 0.4 in the dyspnoea score for the PR group 6 weeks following surgery.

In regard to the QoL global health score 6 weeks following surgery, there was an improvement of 5.1% for the PR group. Furthermore, there was an indication of a 4.7% improvement in the QoL global health score for the PR group compared to the usual care group 6 months following surgery (EE = 4.7). Although it is only 4.7%, the 95% CI indicated that the maximum could be 13 units, which is considered a clinically significant improvement.

## Discussion

The comparative effectiveness of research evaluating the impact of “real world” pulmonary rehabilitation on surgical outcomes is not extensively documented. Randomized controlled trials (RCTs) have strict patient inclusion and exclusion criteria that might restrict the generalisability of the study results. Overall, in this case, matched analysis, the participants in the PR group were matched to the NR group in their demographic characteristics, BMI classification, smoking status, lung function, surgery incision, surgery side, lobe, lung resection technique, comorbidities, and other measures as presented in Table 1. The PPC and LOS showed a trend of being higher in the PR group compared to the NG group, which could be attributed to the PR group having a trend toward a higher prevalence of COPD and greater pack-year histories. Furthermore, this study revealed that the PR group had a reduction of 0.2 days of LOS compared to the usual care group (EE = − 0.20) and that reduction can potentially go up to 1.8 days (95% CI = − 1.8–1.6). Also, in the PPC, the PR group had a reduction of 60% (EE = − 0.60). In the PR group, the QoL domains, including physical functioning, dyspnoea score, and global health score, all improved 6 weeks following surgery, with a maximum improvement of more than 10 units in physical functioning and global health scores 6 weeks and 6 months following surgery, which is considered a clinically significant improvement. Patient participation in the PR program was higher before surgery than after, demonstrating that the intervention is both feasible and acceptable to patients. This increased preoperative engagement highlights the program’s ability to complement patients’ needs effectively during the pre-surgical period.

A preoperative exercise rehabilitation program, pulmonary rehabilitation, has been shown to reduce LOS and PPC and is associated with better QoL. Pulmonary rehabilitation focuses on improving respiratory function and overall health in patients with chronic lung conditions through exercise, education, and support (Spruit et al. 2013). Similarly, prehabilitation, a preparatory intervention aimed at enhancing a patient’s physical and mental fitness before surgery, has demonstrated the potential to improve outcomes and recovery (Drudi et al. 2019; Gravier et al. 2021; Ferreira et al. 2021). Furthermore, pulmonary rehabilitation reduces LOS by enhancing recovery as patients become fitter, and it reduces PPC by improving mobility, enabling more effective coughing, decreasing the likelihood of atelectasis, and enhancing overall breathing (Spruit et al. 2013; Drudi et al. 2019; Gravier et al. 2021). However, due to study heterogeneity, no firm recommendations can be made regarding the optimal exercise modality, delivery method, frequency, or preoperative duration of these interventions. Nonetheless, a preoperative exercise rehabilitation program should be considered, particularly for patients with borderline lung function or limited exercise capacity, to optimize surgical readiness and recovery.

The latter findings correlate with findings from a systemic review that showed prehabilitation was associated with decreased LOS, postoperative complications, improved objective physical functioning, and improved QoL measures in patients undergoing cardiac and vascular procedures (Drudi et al. 2019). Similarly, evidence from another systemic review has shown that prehabilitation improves PPC, exercise capacity, and QoL compared to usual care in patients undergoing non-small lung cancer resection (Gravier et al. 2021). The findings of this study and of those studies presented in the literature suggest that prehabilitation could improve surgical outcomes, including LOS, PPC, and QoL, in patients undergoing cardiac, vascular, and lung cancer procedures (Drudi et al. 2019; Gravier et al. 2021).

Interestingly, this study’s findings contradict findings from an RCT, which showed that prehabilitation had no effect on PPC and LOS in patients scheduled for non-small cell lung cancer resection (Ferreira et al. 2021). However, the same study concurred with this study’s findings in showing the impact of prehabilitation and its association with better QoL, including physical functioning following lung cancer surgery (Ferreira et al. 2021). Nonetheless, it should be noted that this RCT had a short follow-up period, a weakness that may have limited the ability to demonstrate the long-term impact of prehabilitation on QoL (Ferreira et al. 2021).

In addition, this study’s findings oppose those of a recent systemic review that examined prehabilitation for cancer patients and concluded that while some prehabilitation programs showed benefits, many did not significantly improve postoperative outcomes, including postoperative complications, LOS, and QoL compared to usual care (Meneses-Echavez et al. 2023). However, it should be mentioned that the results of this systemic review could be limited by the heterogeneous tools for outcome measurement for the study included in the review (Meneses-Echavez et al. 2023).

Our analyses faced certain limitations typical of observational studies; the results may not be applicable to other major surgeries, different populations, or various settings. Additionally, the uneven availability of PR across sites could introduce site-related bias, as the outcomes observed in patients receiving PR might not be generalisable to those from non-PR sites or to the broader patient population. This limited accessibility could also affect the representativeness of the study sample. Furthermore, the widespread implementation of PR faces multiple barriers, including resource constraints such as funding, infrastructure, and trained personnel, particularly in rural areas, as well as patient adherence challenges such as logistical difficulties, low motivation, and competing medical priorities (Spruit et al. 2013). Inadequate referral pathways, sociocultural factors, and the absence of long-term follow-up further limit accessibility and effectiveness, highlighting the need for targeted interventions to improve enrolment and adherence (Spruit et al. 2013).

While propensity score analysis (PSA) was employed to minimize confounding and improve the comparability of groups, the potential for unmeasured confounding remains a limitation. Certain variables, such as patient motivation, informal caregiver support, or individual clinician practices, may not have been fully accounted for, which could influence both engagement in PR and postoperative outcomes. Despite the use of robust analytical methods, residual confounding cannot be entirely ruled out, and future studies with additional adjustments for such factors may provide further insights into the true impact of PR.

## Conclusion

In summary, engagement in “real-world” pulmonary rehabilitation before surgery appears to result in better patient and clinical outcomes after lung cancer surgery. However, due to the limitations and heterogeneity in study design and patient populations in the literature, the impact of pulmonary rehabilitation before major surgery could not be conclusively determined. Hence, we suggest the need for further rigorous methodological clinical trials to investigate the impact of rehabilitation on perioperative clinical outcomes compared to usual care in major surgeries. Additionally, the public health importance of integrating PR into standard perioperative care should be recognized, including the implementation of a tariff system to promote uptake and accessibility. Future research should also explore what the optimal PR program should be to maximize benefits for patients undergoing major surgery.

## Supplementary Information

Supplementary Material 1.

## Data Availability

Yes, I have research data to declare. The data supporting the results of this manuscript is being saved on a secure server at Queen Elizabeth Hospital under the Data Protection Act (2018) requirements. Upon request through the corresponding author's email, access to data could be provided.

## Abbreviations

PR: Pulmonary rehabilitation
NG: Non-intervention or control group
LOS: Length of stay
PPC: Postoperative pulmonary complications
QoL: Quality of life
PSA: Propensity score analysis
COPD: Chronic obstructive pulmonary disease
MRC: Medical research council
FEV1: Forced expiratory volume in 1 s
DLCO: Diffusing capacity for carbon monoxide
PpoFEV1: Predicted postoperative forced expiratory volume in 1 s
PpoDLCO: Predicted postoperative diffusing capacity for carbon monoxide
VATS: Video-assisted thoracic surgery
MGS: Melbourne Group Scale
EORTC QLQ-C30: European Organisation for Research and Treatment of Cancer Quality of Life Questionnaire (version C30)
EE: Effect estimate
SE: Standard error
CI: Confidence interval
BMI: Body mass index
VATS: Video-assisted thoracic surgery
