# Supplementary material for: Effect of pulmonary rehabilitation on lung cancer surgery outcomes: a matched-case analysis
Source: Perioper Med (Lond). 2025 Mar 25;14:35. doi: 10.1186/s13741-025-00510-2 (PMC11934484; doi:10.1186/s13741-025-00510-2)
Supplement: Supplementary file 1 — Supplementary Material 1. [file 13741_2025_510_MOESM1_ESM.docx]

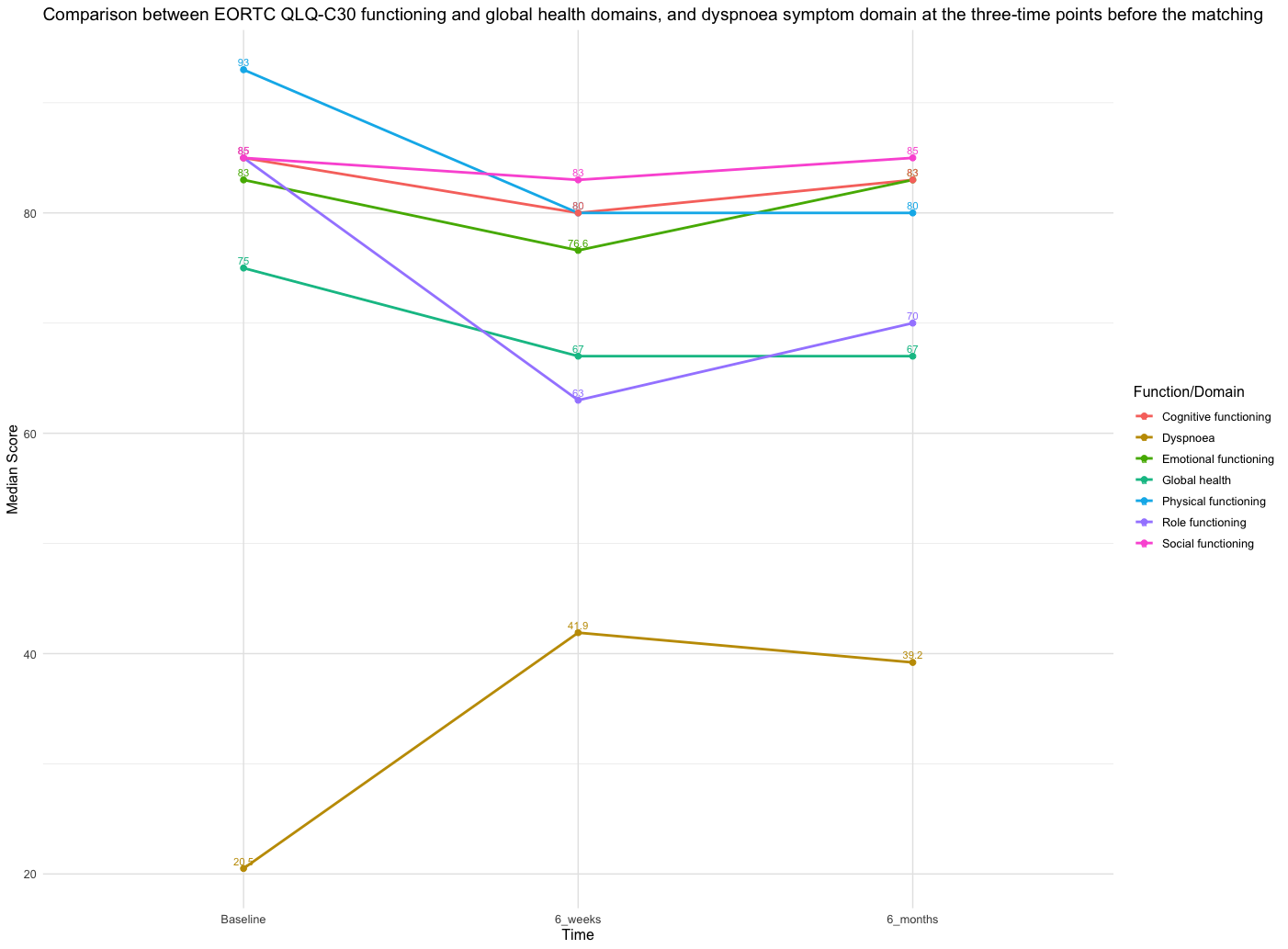


**Figure (3): Comparison between EORTC QLQ-C30 functioning and global health domains, and dyspnoea symptom domain**


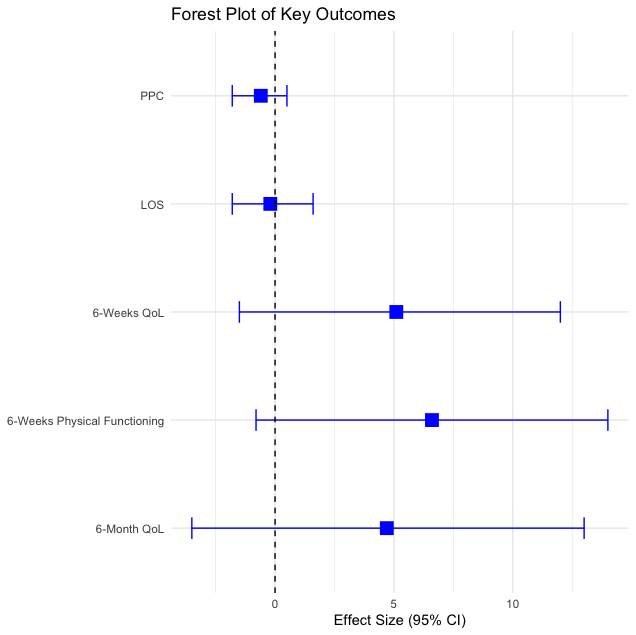


**Figure (4): Forest plot of key outcomes**
